# Case Report: MIS-C precipitating DKA and renal failure in a morbidly obese male with type 2 diabetes mellitus

**DOI:** 10.3389/fped.2026.1727955

**Published:** 2026-03-17

**Authors:** John Tumberger, Vincent Czerwinski, Francesca Pérez Marquès, Travis R. Langner, Hammad A. Ganatra, Shawn B. Sood

**Affiliations:** 1 Division of Pediatric Critical Care, University of Kansas Medical Center, Kansas City, KS, United States; 2 University of Kansas School of Medicine, Kansas City, KS, United States

**Keywords:** obesity, DKA (diabetic ketoacidosis), MIS-C (multisystem inflammatory syndrome in children), T2D (type 2 diabetes), pediatric, critical care

## Abstract

Multisystem inflammatory syndrome in children (MIS-C) is a recently described systemic inflammatory sequelae of SARS-CoV-2 infection. Diagnostic criteria for MIS-C includes age <21, fever (>38°C), laboratory evidence of inflammation, clinically severe illness requiring hospitalization with multisystem (≥2) organ involvement, Evidence of current or recent SARS-CoV-2 infection or exposure, and has no alternative explanation for their symptoms. We present a 15-year-old, morbidly obese male whose diagnosis of MIS-C was obfuscated by diabetic ketoacidosis (DKA). This case highlights the increased risk of development of MIS-C in obese children, variability of presentation, and contributes to the body of research demonstrating endocrinologic complications of MIS-C.

## Introduction

MIS-C is a systemic inflammatory sequela of SARS-CoV-2 infection which is diagnosed in patients less than 21 years who present with fever laboratory evidence of inflammation, clinically severe illness requiring hospitalization with multisystem (≥2) organ involvement, evidence of current or recent SARS-CoV-2 infection or exposure, and has no alternative explanation for their symptoms (1). In the following case, a 15-year-old, morbidly obese male with known type 2 diabetes (T2DM) on insulin, initially presented with DKA. Despite initial normalization of glucose, plasma bicarbonate, and ketonuria, the patient clinically deteriorated and developed renal failure, encephalopathy, disseminated intravascular coagulation, shock, and respiratory failure. This case illustrates a rare presentation of MIS-C manifesting as DKA in a morbidly obese patient with preexisting type 2 diabetes mellitus, highlighting novel aspects of disease interplay and severity.

## Case presentation

A 15-year-old male with a past medical history of severe obesity (258.6 kg, BMI 75), T2DM on insulin, and hypertension presented to a regional medical center with a two-day history of nausea and emesis. Vitals were remarkable for tachycardia (heart rate of 138), tachypnea (respiratory rate 42) and a blood pressure of 138/83. Exam was significant for a GCS of 14, Kussmaul respirations and several open sacral chafing wounds. Initial laboratory evaluation was remarkable for leukocytosis (15.5e3 cells/μL) with a neutrophilic predominance, hyponatremia (131 mmol/L), hyperglycemia (759 mg/dL), and an elevated creatinine (1.86 mg/dL). Urinalysis featured 2 + protein, 2 + hemoglobin, 3 + glucose, and 3 + ketones. Arterial blood gas demonstrated acidosis (pH 6.96), decreased carbon dioxide (pCO2 10 mmHg) and an arterial bicarbonate (2 mEq/L). Beta-hydroxybutyrate was elevated beyond the level of quantification, and hemoglobin A1c was 14.7. Respiratory viral panel, troponin, thyroid-stimulating hormone, electrocardiogram, and chest x-ray were all unremarkable. T1DM serology was negative. Laboratory profile across selected times is presented in Table 1.

**Table 1:** Laboratory values from initial presentation and arrival to our institution.

Parameter	Day 0	Day 1
Vitals		
BP:	138/83	74/30
Pulse:	138	149
RR:	42	18
Temp (F):	91.5	99.9
Blood:		
WBC (3.6–9.1 10E9/L)	15.5	13.0
Hemoglobin (12.8–16.0 g/dL)	14.4	11.4
Platelets (150–450 10E9/L)	393	234
Neutrophils % (48%–85%)	85.6	73
Lymphocytes % (14%–43%)	4.8	2
Chemistry:		
Sodium (136–145 mmol/L)	131	140
Potassium (3.6–4.9 mmol/L)	4.2	3.8
Chloride (99–111 mmol/L)	101	115
HCO3 (20–36 mmol/L)	<10	13
Glucose (74–106 mg/dL)	759	145
BUN (6–20 mg/dL)	12	36
Creatinine (0.50–1.00 mg/dL)	1.86	4.54
Total Bilirubin (<=1.2 mg/dL)	0.4	0.5
Alkaline Phosphatase (100–390 U/L)	207	120
ALT (10–46 U/L)	30	19
AST (15–45 U/L)	34	15
Lactic acid (0.70–2.00 mmol/L)	1.31	0.8
Urine:		
Color	Yellow	Amber
Appearance	Clear	2+ turbidity
Specific Gravity (1.003–1.030)	1.027	1.024
pH (5.0–8.0)	5.5	5
Leukocyte Esterase	Negative	Negative
Nitrites	Negative	Negative
Protein	2+	3+
Glucose	3+	1+
Ketones	3+	Negative
Hemoglobin	2+	3+

Values in red indicate deviation from the normal range.

Initial management included intravenous hydration, insulin infusion, and frequent electrolyte laboratory monitoring. Over the first twenty-four hours of admission, insulin was steadily increased to 16 units/h leading to improvement of ketonuria, normalization of anion gap, and a glucose nadir of 105 mg/dL. Despite resolving DKA, the patient had progressively diminishing urine output eventually becoming anuric with a worsening creatinine (1.8 mg/dL at admission to >4 mg/dL on day 2). Renal and bladder ultrasound demonstrated minimal urine in the bladder without hydronephrosis. Furthermore, the patient had worsening hyperpnea, new onset hypoxia despite BIPAP (IPAP 12/ EPAP 10/ FiO2 70%), decreased level of consciousness, and leukocytes in his urine.

Given the patient's worsening kidney failure, respiratory failure, and altered mental status despite treatment of DKA he was transferred to an academic tertiary pediatric intensive care unit (PICU). In transit, the patient became obtunded and hypotensive (74/30 mmHg). Upon arrival, the patient was sedated, intubated, and central access was obtained. Epinephrine and Norepinephrine were initiated secondary to hemodynamic instability. Head CT revealed no cerebral edema. After failing a diuretic load, the patient was initiated on continuous renal replacement therapy (CRRT) to manage fluid overload, electrolyte disturbances, and acute renal failure.

**Figure 1:**
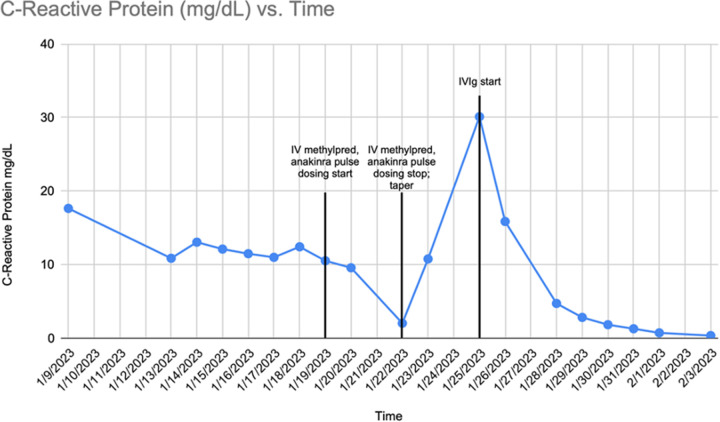
C-Reactive Protein vs. Time.

**Figure 2:**
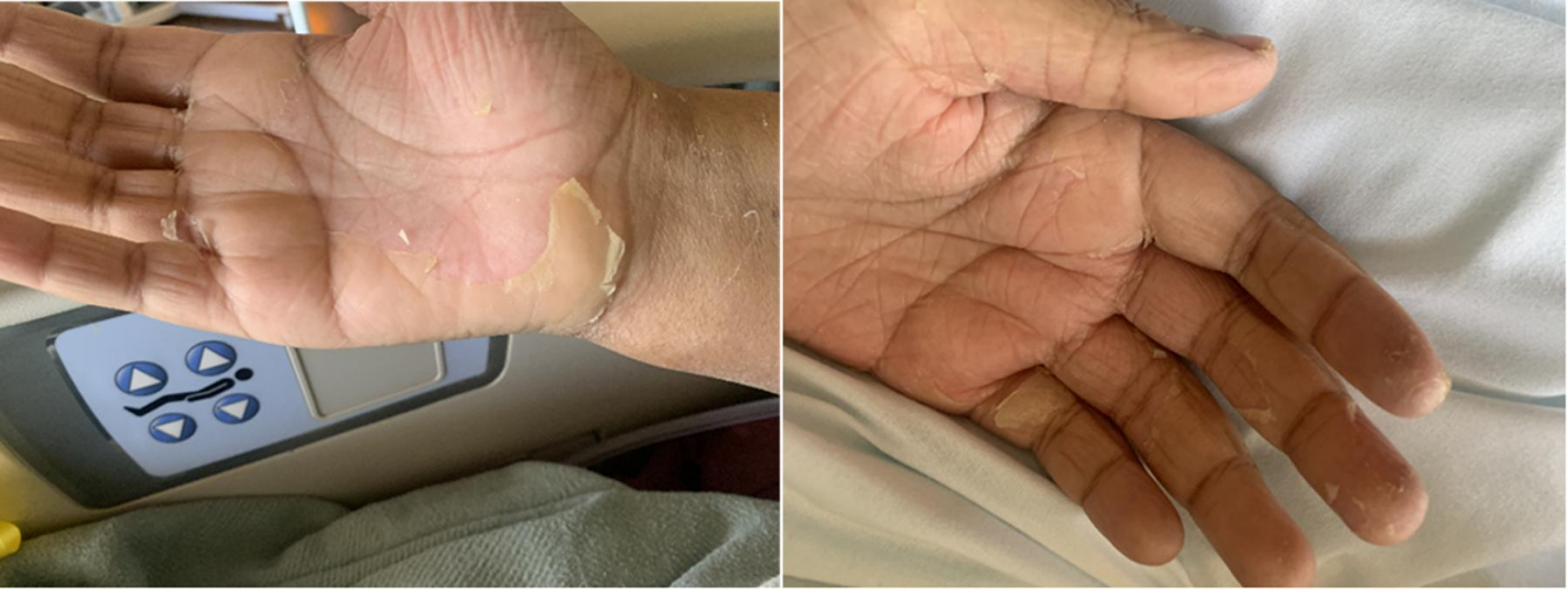
Desquamating right, left hands.

Over the next fourteen days, the patient continued to experience oliguria and hypotension requiring inotropic support. The patient was initiated on a therapeutic heparin dose as several of his central venous access sites developed occlusive thrombi (d-dimer max of 24197 ng/mL) (Table 2). ESR and CRP were consistently elevated in these first two weeks with negative blood culture growth (Figure 1). The persistent, systemic, inflammatory presentation without clear etiology prompted consideration of rheumatologic entities. On hospital day fourteen, a SARS-CoV-2 antibody test was performed which returned positive, and the patient was initiated on anakinra (IL-1 receptor antagonist) and methylprednisolone (corticosteroid). IVIg was then initiated. Over the following week, the patient's pressor requirements steadily decreased, end-organ function improved, and sedation was successfully weaned. Renal function and inflammatory markers improved, and the patient was switched from CRRT to hemodialysis (HD). On hospital day twenty-one the patient was successfully extubated. Physical exam at that time was significant for the development of a desquamating rash on the palms of his hands Figure 2. Thirty days into the hospital course the patient was transferred to the pediatric floor. The patent was eventually discharged to home. Follow up was completed at an outside facility and additional information about long term outcomes is not available.

**Table 2:** Additional laboratory testing.

Parameter	Value
Additional testing:	
High sensitivity troponin (<=53 pg/mL)	5
Albumin (g/dL)	2.7
Beta-hydroxybutyrate (<2.8 mg/dL)	>46.8
Hemoglobin A1C (<5.7%)	14.70%
D-Dimer (0.00–0.50 ug/mL FEU's)	3.60
Fibrinogen (200–393 mg/dL)	677
Respiratory viral panel	All negative
Ferritin (12–300 ng/mL)	622
Procalcitonin (0.10–0.49 ng/mL)	3.95
COVID-19 Spike RBD, Ab (<0.80 U/mL)	>250
T1DM Testing:	
Insulin antibody, esoterix (<5.0 uU/mL)	6.8
IgA Quantitative (52–221 mg/dL)	<5
anti-GAD (0.0–5.0 U/mL)	<5.0
Zinc transporter 8 autoantibodies (<15 U/mL)	<15
IA2 autoantibody (<7.5 U/mL)	<7.5
anti t-Transglutaminase IgA (0 3 U/mL)	<2

Values in red indicate deviation from the normal range.

## Discussion

MIS-C is a systemic, inflammatory condition characterized by multiple organ failure and laboratory findings of inflammation which is preceded by SARS-CoV-2 infection. Evidence supporting the diagnosis of MIS-C include age <21, fever (>38°C), laboratory evidence of inflammation, clinically severe illness requiring hospitalization with multisystem (≥2) organ involvement, evidence of current or recent SARS-CoV-2 infection or exposure, elevated D-dimer, anemia, elevated ferritin, elevated fibrinogen, low albumin, history of upper respiratory infection preceding admission, development of desquamating rash, with a SARS-CoV-2 antibody level >250 U/mL (1–3).

As a systemic condition, the symptoms of MIS-C are variable. A systematic review of MIS-C identified gastrointestinal complaints as the most common presenting symptoms (71%) (3). Cardiac dysfunction is a hallmark of MIS-C, evidenced by proBNP elevation in approximately 77% of cases and shock necessitating inotropic support in 63% (4). Other hyper-inflammatory syndromes affecting children such as Kawasaki disease, Hemophagocytic Lymphohistiocytosis (HLH), and Macrophage Activation Syndrome (MAS) have overlapping features with MIS-C such as persistent fever and laboratory evidence of inflammation (5). Furthermore, treatment strategies such as high-dose corticosteroids and immunomodulatory therapies such as IVIg and anakinra comprise a shared treatment approach (4).

Our case represents the largest reported BMI of any MIS-C patient (6, 7). Obesity is a well-established risk factor for both the development and severity of MIS-C. In descriptions of MIS-C, overweight and obesity are overrepresented, with up to 50% of MIS-C patients falling into one of these categories (8). A large systematic review showed that children with obesity are significantly more likely to develop MIS-C following SARS-CoV-2 infection compared to their normal-weight peers, with hazard ratios as high as 2.2 (6, 7, 9). Obesity is associated with immune dysfunction leading to increased sepsis mortality (10). Similarly, it is possible that obesity-related immunomodulation exacerbates the inflammatory pathogenesis of MIS-C. However, it is also plausible that the association in mortality is due to obesity exacerbating morbid conditions such as respiratory and circulatory failure.

Severe renal failure to the extent seen in our patient represents a relatively uncommon finding in MIS-C. While it is possible that the renal injury was related to MIS-C, it could also be attributed to other factors in this patient's clinical course, such as hypovolemia secondary to DKA, cardiovascular shock and associated renal hypoperfusion. Lastly, the patient's mental status changes represented a diagnostic conundrum, as DKA related hyperglycemia or cerebral edema could both explain worsening mental status, but CT imaging did not reveal cerebral edema, and his neurological symptoms persisted following correction of DKA. This suggests that additional etiologies were likely at play. MIS-C has been reported to produce severe neurological manifestations such as encephalopathy and seizures (11), and it is possible that our patient's mental status was a manifestation of MIS-C. We also recognize that uremic encephalopathy in the setting of anuric renal failure could have contributed to his depressed mental status.

The rapid improvement of multiorgan failure following anakinra and methylprednisolone further supported the diagnosis of MIS-C (12). Furthermore, our case highlights that MIS-C may not feature COVID-19 PCR positivity, and the workup of a multisystem inflammatory condition requires COVID-19 serologic testing.

Finally, DKA as a presenting symptom is a very uncommon finding in MIS-C. There is a small body of evidence linking MIS-C and new-onset type 1 diabetes (T1DM) presenting in DKA (13). These cases generally featured an underweight individual with serologically proven T1DM. To date, only 2 cases of T2DM presenting with DKA and MIS-C has been reported (14, 15). In the other cases, DKA was the initial presenting symptom without serologic evidence of T1DM. In contrast, our case was an obese, known T2DM patient who had a new episode of DKA. Taken together, these cases may suggest that COVID-19 infection could lead to impaired beta cell functionality. Alternatively, a case-series of two children who developed antibody-negative diabetes who presented in DKA after treatment of Kawasaki disease (16) suggests a relationship between massive, acute inflammation and pancreatic dysfunction. Additional inquiry is required to elucidate the mechanism behind SARS-CoV-2 leading to beta cell dysfunction.

## Data Availability

The original contributions presented in the study are included in the article/Supplementary Material, further inquiries can be directed to the corresponding author.
